# The clinical manifestations and treatment of male breast cancer: a report of three cases

**DOI:** 10.1186/s40792-015-0103-8

**Published:** 2015-10-05

**Authors:** Shuji Suehiro, Miyuki Abe, Yohei Takumi, Takafumi Hashimoto, Mirei Kamei, Atsushi Osoegawa, Michiyo Miyawaki, Kenji Sugio

**Affiliations:** Department of Thoracic and Breast Surgery, Faculty of Medicine, Oita University, 1-1 Idaigaoka Hasama-machi, Yufu, Oita 879-5593 Japan

**Keywords:** Male breast cancer, Mastectomy, Adjuvant therapy

## Abstract

Male breast cancer is an extremely rare malignancy. We treated three male breast cancer patients. All three patients showed clinical N0 and received sentinel lymph node biopsy. Because the sentinel lymph node was positive for metastasis in one patient, a total mastectomy with axillary lymph node dissection was performed. The other two patients were negative for sentinel lymph node metastasis, and a simple mastectomy was performed. Two of the patients were postoperatively treated with tamoxifen; another patient was treated with adjuvant chemotherapy using taxotere and cyclophosphamide before tamoxifen. There was no recurrence in any of the three patients during an average follow-up period of 56.7 months (range 11.8–80.3). A sentinel lymph node biopsy is recommended for node staging in both male and female breast cancer patients as it is associated with a lower incidence of complications.

## Background

Male breast cancer (MBC) is an extremely rare malignancy which accounts for 1 % of all breast cancers and 0.1 % of all male cancers [1]. In spite of the low incidence of MBC, it is associated with a higher mortality than female breast cancer (FBC), due to the more advanced staging of patients at the time diagnosis [2]. In 2013, only 82 of the 13,230 patients in Japan who died as a result of breast cancer were males [3]. Because of the rarity of MBC, its etiology is less clear than FBC. The management of patients with MBC generally depends on the information from the diagnosis and the processes and treatments of FBC. However, in contrast to FBC patients who are more often treated with breast-conserving surgery, most MBC patients are treated with simple mastectomy with axillary lymph node dissection (ALND) or sentinel lymph node biopsy (SLNB) [4–6]. We herein report three MBC cases with information on the diagnostic procedures and management, including the performance of SLNB.

## Case presentation

SLNB: At the day before operation, sentinel lymphoscintigraphy was performed, with the injection of 99 m Tc (0.2 ml) using a Techne Phytate Kit. Sentinel lymph nodes (SLNs) were detected with a hand-held gamma probe (Navigator GPS, Sheen man, Osaka, Japan). When an excisional biopsy had already been carried out, subdermal injection was performed near the scar. Planar scintigraphic scans of the involved breast and the axillary region in the anterior and anterior-oblique projections were carried out after 4 h of injection.

Under general anesthesia, indocyanine green (1 ml) was injected into the periareolar skin and periphery of tumor for SLNB by the dye method. SLNs were traced by the Navigator GPS and confirmed by the presence of a radioisotope and dye. The pathological diagnosis of the resected SLNs was performed during surgery.

## Case 1

**Fig. 1:**
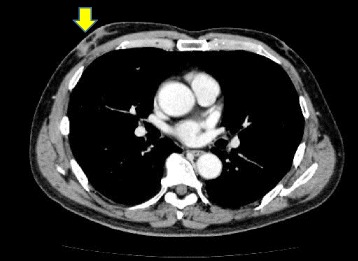
A preoperative chest CT scan showing the incisional biopsy scar (*arrow*) of the right breast carcinoma

A 64-year-old man presented to his family doctor complaining of a tumor in his right breast that had been present for 2 years. A CT showed a round tumor of 10 mm in diameter under the nipple. There was no evidence of axillary lymph node swelling or metastatic disease (Fig. 1). Mammography (MMG) and ultrasonography (US) were not performed. An excisional biopsy was performed, which revealed an invasive ductal carcinoma with a close margin (<5 mm). He was introduced to our hospital for operation. The clinical diagnosis was cT1cN0M0, stage I. Because the pathological margin of the excisional biopsy was close (<5 mm), we explained to the merits and demerits of the different procedures (including partial mastectomy or total mastectomy), and an operation was selected. In our breast surgery department, MRI of the breast is usually performed for patients who intend to undergo partial mastectomy. We therefore performed total mastectomy and SLNB.

On the day before the operation, sentinel lymphoscintigraphy was performed. The SLNs were indicated by the combination of a radioisotope and blue dye. No metastasis was found in the intraoperative examination of frozen sections of four SLNs. After the SLNB, a total mastectomy of the right breast was performed. In the pathological examination of the SLNs, an H&E staining showed no metastasis. The tumor was diagnosed as an invasive ductal carcinoma (histological grade 1, estrogen receptor (ER)-positive, progesterone receptor (PR)-positive, HER2 0). The final diagnosis was pT1bN0M0, stage I. Because of the ER and PgR positivity of the tumor, the patient was treated with tamoxifen (20 mg) for 5 years (Table 1). The patient did not suffer any complications and remains alive without any recurrent disease at 80.3 months after the surgery.

**Table 1:** The clinical and pathological characteristics of three male breast cancer patients

Case	Age	Tumor size (mm)	T	N	M	Stage	Histology	Nuclear grade	ER	PgR	HER2	Ki67	Adjuvant therapy
1	64	10	1	0	0	I	Solid-tubular carcinoma	1	+	+	0	Unknown	Tamoxifen 20 mg
2	64	16	1	0	0	I	Solid-tubular carcinoma	1	+	+	0	Unknown	Tamoxifen 20 mg
3	69	24	2	1	0	IIB	Scirrhous carcinoma	3	+	+	0	21.2 %	Taxotere (75 mg/m^2^) and cyclophosphamide (600 mg/m^2^)
													Tamoxifen (20 mg)

*T* tumor, *N lymph* node, *M* metastasis

## Case 2

**Fig. 2:**
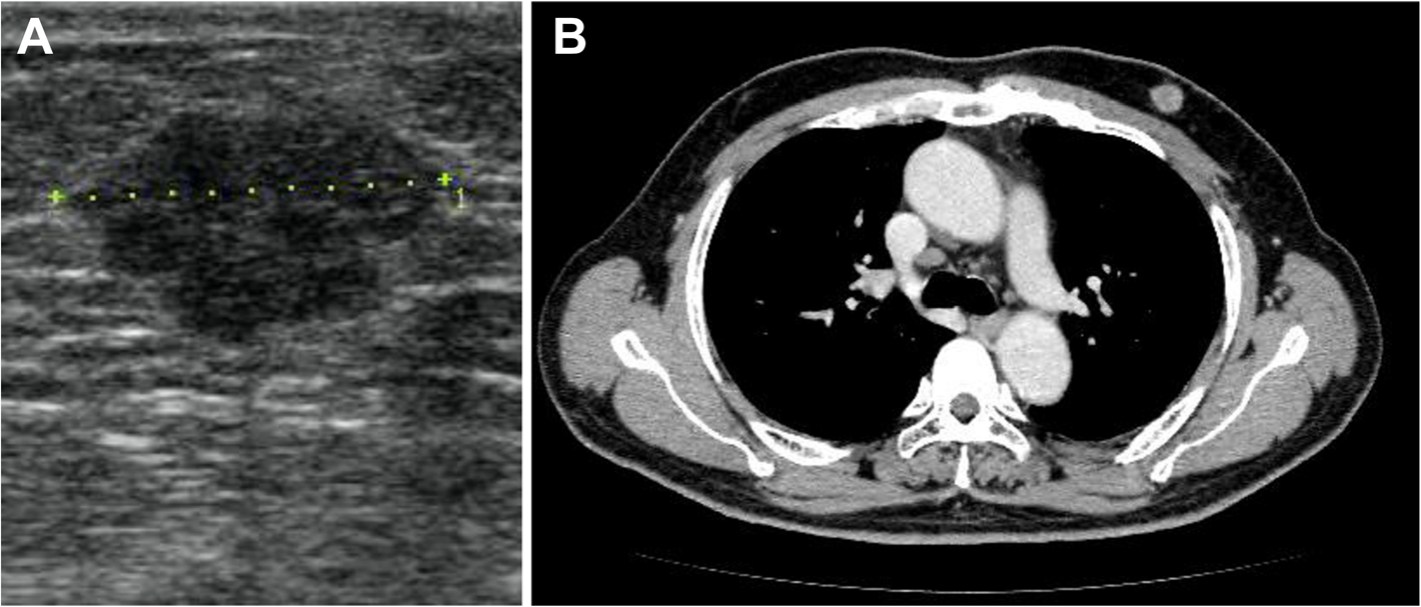
**a** A preoperative US scan showing a round mass of 16 mm in diameter in the left subareolar area. **b** A preoperative chest CT scan showing a round tumor of 16 mm in diameter in the left subareolar area

A 64-year-old man visited to our hospital complaining of a tumor in his left breast that had been present for at least 3 months. A US scan showed a well-defined round tumor measuring 16 mm in diameter under the nipple (Fig. 2a). A cytological analysis of a fine needle aspiration biopsy specimen suggested malignancy. A CT scan showed a round tumor of 16 mm in diameter under the nipple without axillary lymph node swelling or distant metastasis (Fig. 2b). MMG were not performed. The clinical diagnosis was cT1cN0M0, stage I. On the day before the operation, sentinel lymphoscintigraphy was performed. Under general anesthesia, SLNB was performed by the radioisotope and dye method. The pathological diagnosis of the resected SLNs was conducted during surgery. No metastasis was found in the intraoperative examination of frozen sections of the two SLNs. After the SLNB, a total mastectomy of the left breast was performed. The tumor was diagnosed as an invasive ductal carcinoma (histological grade 1, ER-positive, PR-positive, HER2 0, Ki67 index 22.3 %). The pathological examination of the SLNs showed no signs of metastasis. The final diagnosis was pT1cN0M0, stage I. Because of the ER and PgR positivity of the tumor, the patient was treated with tamoxifen (20 mg) for 5 years. The patient did not suffer any complications and remains alive without any recurrent disease at 78 months after surgery.

## Case 3

**Fig. 3:**
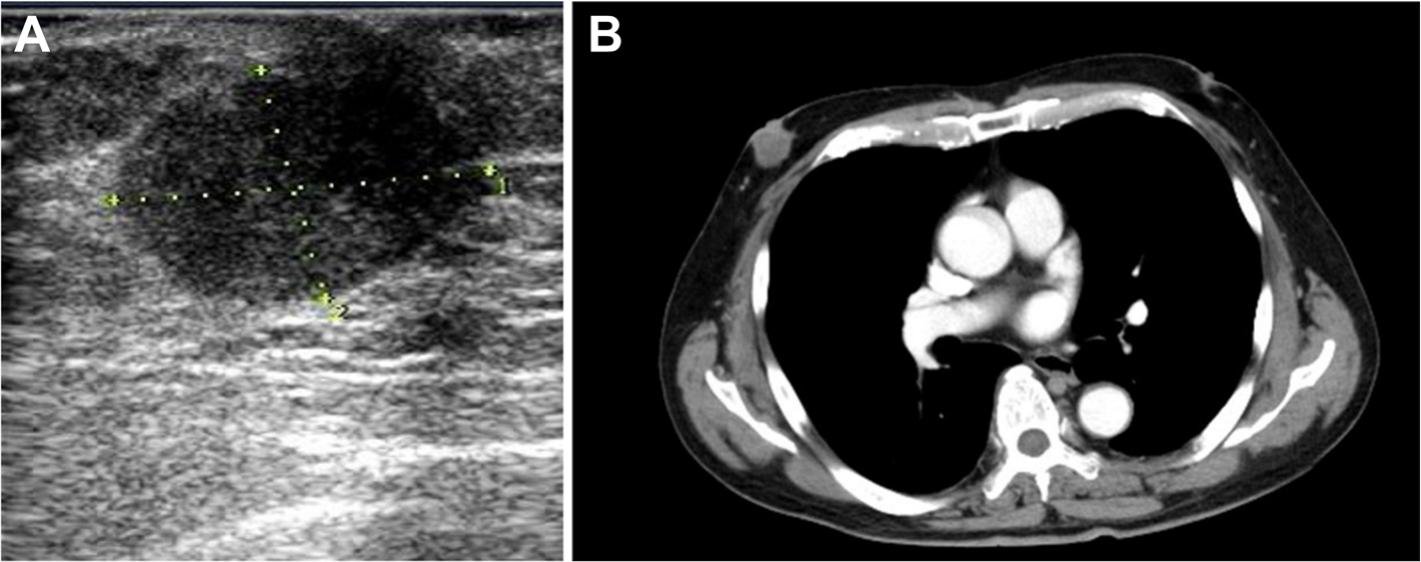
**a** A preoperative US scan showing a round mass of 24 mm in diameter in the right subareolar area. **b** A preoperative chest CT scan showing a round tumor of 24 mm in diameter in the right subareolar area

A 69-year-old man presented to his family doctor complaining of a tumor in his right breast that he noticed a few days before his presentation. A US scan showed a well-defined round tumor of 25 mm in diameter under the nipple (Fig. 3a). A core needle biopsy suggested invasive ductal carcinoma. A CT scan showed a tumor of 24 mm in diameter under the nipple without axillary lymph node swelling or distant metastasis (Fig. 3b). He was referred to our hospital for surgery. The clinical diagnosis was cT2N0M0, stage IIA. On the day before operation, sentinel lymphoscintigraphy was performed. SLNB was performed by the radioisotope and dye method. A pathological examination of the three removed SLNs was conducted pathological during surgery, which showed metastasis in one SLN. After SLNB, a total mastectomy of the left breast was performed with level II axillary lymph node dissection. The tumor was diagnosed as an invasive ductal carcinoma (histological grade 3, ER-positive, PR-positive HER2 score 0, Ki67 index 21 %). A pathological examination showed metastasis in 2 of 15 axillary lymph nodes. The final diagnosis was pT2N1M0, stage IIB. Adjuvant chemotherapy, consisting of taxotere (75 mg/m^2^, 120 mg/body), and cyclophosphamide (600 mg/m^2^, 1000 mg/body) was administered in four courses following the standard treatment guidelines for FBC. Adjuvant chemotherapy without anthracycline was administered because of the patient’s hypertension. After chemotherapy, he was treated with tamoxifen (20 mg) due to the ER and PgR positivity of the tumor. The patient experienced axillary paresthesia, a decreased range of motion of the shoulder and arm, but has experienced no recurrence in the 10 months since surgery.

### Discussion

MBC is a rare malignant tumor, which accounts for less than 1 % of breast cancer cases [1]. The median age at diagnosis among MBC patients is 67–71 years, slightly older than the median age of 61 years in female patients [7, 8]. Because MBC tumors are likely to express ER and PgR, the use of adjuvant hormonal therapy is beneficial [5]. Some reports have suggested that the frequency of high expression human epidermal growth factor 2 (HER2) is low [9–11]. In this study, all three patients showed ER-positive and HER2 scores of 0 and were treated with tamoxifen. The side effects of tamoxifen include weight gain, hot flashes, decreased sexual desire or ability, and mood swings. The side effects occur more frequently in MBC patients than in FBC patients [12]. Our patients reported no side effects in relation to the administration of tamoxifen during the follow-up period. Because of the rarity of MBC, the etiology of MBC is less clear than the etiology of FBC. The management of patients with MBC generally depends on the information from the diagnosis process and the treatments that are applied to patients with FBC. We are currently continuing to treat case 3 with tamoxifen (20 mg), while being careful to identify the occurrence of any adverse events.

The prognosis of MBC had been reported to be worse than that of FBC. The reason for the lower overall survival associated with MBC is the higher age of susceptibility and the higher rate of cases who are diagnosed with advanced stage MBC [2]. When adjusted for the expected survival rate of the US population, race, gender, and age, no differences were found in the 5-year survival rate for MBC and FBC. The 5-year survival rate at each stage of MBC vs. FBC is 96 vs. 99 % (stage I), 84 vs. 84 % (stage II), 52 vs. 55 % (stage III), and 24 vs. 18 % (stage IV), respectively [8]. Due to the lack of knowledge about MBC, patients may take longer to seek medical treatment after noticing, which may result in an increase in the tumor diameter [13]. Cases of MBC are often diagnosed due to the presence of a breast tumor [14].

In this case report, we performed mastectomy for all three patients. We usually explained the merits and demerits of the different procedures (including partial mastectomy or total mastectomy) to each patient. Although there were no statistically significant differences in the survival rates of the patients who underwent total mastectomy and partial mastectomy followed by irradiation, the local recurrence rate was higher in the patients who underwent partial mastectomy. To date, no studies have shown the superiority of the breast-conserving surgery (BCS) for male patients. As a result, we select mastectomy with SLNB for our male patients. A simple mastectomy is the most common surgery for MBC patients, because it is associated with a lower rate of local recurrence rate in comparison to BCS [6]. Almost all male patients are treated with mastectomy, due to small volume of breast tissue in comparison to females. BCS for FBC patients is the standard therapy because it is associated with a lower level of mental anguish. In contrast, male patients show less mental anguish in association with breast loss. Furthermore, radiotherapy is necessary for BCS, which involves a long treatment period and increased medical costs. It is therefore reasonable to enforce mastectomy in MBC patients. There are no reports of differences in the incidence of postoperative complications in MBC and FBC patients. Generally, the rate of males who engage in manual labor is higher than that in females; therefore, the influence ALND is higher in MBC patients than in FBC patients.

ALND may be associated with scarring, axillary paresthesia, a decreased range of motion of the shoulder and arm, and lymphedema [15]. Although the usefulness of SLNB in female patients has been proven [16], it is difficult to establish the usefulness and safety of SLNB in MBC patients, but it is important to accumulate data. The patient who underwent ALND showed axillary paresthesia, a decreased range of motion of the shoulder and arm, while the other two patients who only underwent SLNB did not show any complications. As in FBC patients, SLNB is recommended for staging the axilla in MBC patients and is associated with fewer complications than ALND. Overgaard et al. suggested that the survival benefit after postmastectomy RT was substantial and similar in patients with 1–3 and 4+ positive lymph nodes in a subgroup analysis of randomized trials [17]. In case 3, we explained the potential survival benefit associated with radiation therapy after chemotherapy, but the patient refused to undergo radiation therapy because of the long-term nature of the treatment.

## Conclusions

Although MBC is rare disease, a clinical trial is necessary to establish a standard treatment for MBC. In this study, three male patients with breast cancer were treated the same as female patients, and all patients remain alive without recurrence.

## Consent

We routinely obtained general consent from every patient for using their clinical data before surgery. Written informed consent was not obtained from the patient for publication of this case report because this report is just a retrospective case report without additional invasive examinations or treatments for the study.

## Abbreviations

ALND: axillary lymph node dissection
BCS: breast-conserving surgery
ER: estrogen receptor
FBC: female breast cancer
HER2: human epidermal growth factor 2
MBC: male breast cancer
PgR: progesterone receptor
SLN: sentinel lymph node
SLNB: sentinel lymph node biopsy
